# Discrepant Spatiotemporal Characteristics of Gait Impairments in Thalamic Infarction Patients

**DOI:** 10.1002/brb3.70582

**Published:** 2025-05-26

**Authors:** Congjun Li, Chen Ye, Rui Sun, Ruosu Pan, Shuai Jiang, Yuyign Yan, Tang Yang, Le Cao, Hang Wang, Youjie Wang, Junfeng Liu, Wendan Tao, Bo Wu

**Affiliations:** ^1^ Department of Neurology West China Hospital, Sichuan University Chengdu China; ^2^ Center of Cerebrovascular Diseases West China Hospital, Sichuan University Chengdu China

**Keywords:** Cerebral small vessel disease, Fine motor disorder, Gait disturbance, Ischemic stroke, ReadyGo, Thalamic infarction

## Abstract

**Background and Objectives:**

Thalamic infarction (TI) can lead to gait disturbances (GDs), even in the absence of significant motor impairments. Understanding the characteristics of GDs in TI patients is crucial for developing targeted rehabilitation strategies. Nonetheless, very little is known about the detailed gait changes in TI patients. This study aimed to investigate and characterize these parameters in TI patients.

**Methods:**

Ninety participants, including 45 subacute TI patients and 45 age‐sex‐matched healthy controls, were cross‐sectionally and consecutively included from West China Hospital, Sichuan University. A detailed set of spatiotemporal gait analyses was performed with forty‐one parameters as output, evaluated using the “ReadyGo” three‐dimensional motion balance testing system. Additionally, we analyzed the correlation between cerebral small vessel disease (CSVD) and gait parameters in TI and healthy controls (HC) and performed a Fisher z‐test to determine whether there was a significant difference.

**Results:**

Variability, stride length, stride speed, and swing velocity significantly differed in the affected and unaffected sides of TI patients. TI patients exhibited differences in thirty‐eight gait parameters compared to controls. Coordination analysis revealed impairments in the timed up and go test, with longer total time, turn time, stand‐up time, and reduced stride speed. Additionally, deficits were noted in the Heel‐Knee‐Shin test and Finger‐Nose test. However, no differences were found in Romberg's test. Balance assessment showed variations in sit time, torso rocking degree, torso forward roll degree, and walking speed. The correlation between gait parameters and CSVD in TI and HC is presented. Additionally, it was found that total burden leads to a decrease in step width in TI patients and increases trunk sway degree in the tandem stance test in TI.

**Conclusion:**

This study demonstrates distinct spatiotemporal gait impairment patterns, coordination, and balance deficits in TI patients. Additionally, our findings suggest that the mechanisms underlying GDs may differ between TI patients and HC in relation to CSVD. These findings emphasize the need for personalized rehabilitation strategies to target these specific GDs in TI patients.

## Graphical abstract

This study utilizes ReadyGo for the quantitative analysis of gait in TI patients and HC without obvious motor impairments, comparing differences between the unaffected and affected sides of TI patients, as well as between TI patients and HC, and reports the baseline gait parameters of TI patients.

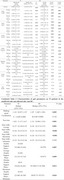

## 1 Introduction

TI is a subtype of ischemic stroke accounting for 3.1% to 8% of all cases (Del Brutto et al. 1993; Saez de Ocariz et al. 1996; Feigin et al. 2022). Given the thalamus's crucial role in sensory‐motor integration, TI presents with unique GDs that differ from those seen in basal ganglia lesions, such as ataxia and axial postural instability syndromes (Masdeu 2016). Damage to thalamo‐cortical circuits—particularly the ventrolateral nucleus involved in motor regulation—may trigger distinct compensatory gait strategies (Verlinden et al. 2016). However, most studies on TI‐related gait have been grouped under lacunar infarctions within the broader CSVD spectrum (de Laat et al. 2010; Sharma et al. 2023), and typically report only general features like reduced step length and gait speed, with few direct comparisons to other chronic cerebrovascular disorders.

CSVD is a chronic pathology involving small intracranial arteries, capillaries, and venules, and is highly prevalent in the elderly. It is a recognized contributor to GD (Liu et al. 2025; Blumen et al. 2023) CSVD‐related gait changes are linked to disruption of cortico‐subcortical circuits, including the frontal‐basal ganglia loop, and are often accompanied by compensatory prefrontal overactivation during locomotion (Sharma et al. 2023; Parihar et al. 2013; Cai et al. 2025). Notably, many TI patients concurrently exhibit CSVD burden. Although both conditions share microvascular pathology, their injury patterns differ: TI involves focal thalamo‐cortical disconnection, while CSVD causes diffuse white matter damage, leading to divergent gait compensation mechanisms.

Traditional tools such as the Tinetti test and timed up and go (TUG) test lack the sensitivity to detect subtle gait abnormalities, particularly in CSVD and TI (Virmani et al. 2018; Ansai et al. 2018)

Based on the multidimensional model proposed by Lord et al. (Lord et al. 2013), modern gait analysis frameworks assess five key domains: pace, rhythm, variability, asymmetry, and postural control. McArdle et al. confirmed its utility in distinguishing neurodegenerative diseases (Elasfar et al. 2025). However, device variability—e.g., pressure‐sensitive walkways vs inertial sensors—limits cross‐study comparability. While pressure systems offer high spatial accuracy, they cannot assess postural dynamics (Giacomozzi et al. 2014); wearables like APDM are portable but prone to signal drift, compromising reliability in variability and asymmetry detection (Mancini and Horak 2016).

To overcome these limitations, we employed the AI‐driven gait analysis system ReadyGo (Mo et al. 2024; Chen et al. 2024), which utilizes deep learning‐based skeletal tracking to extract spatiotemporal parameters from video data without wearable sensors. This study aims to comprehensively quantify gait characteristics—including pace, balance, and coordination—in TI patients and explore their associations with CSVD imaging markers, providing a foundation for individualized rehabilitation strategies.

## 2 Method

### 2.1 Study Participants and Clinical Data Collection

From October 2021 to October 2024, patients who experienced an initial unilateral thalamic ischemic stroke were continuously enrolled in the Department of Neurology of West China Hospital, Sichuan University. Concurrently, individuals without any pre‐existing neurological conditions or vascular risks, who were matched in terms of age and sex, were recruited as healthy control participants from willing residents within the local communities. Written informed consent was obtained from each participant or their legal guardians, and approval of our project was obtained from the Ethics Committee of West China Hospital of Sichuan University [No. 2020 (922)].

Patients were included in the study if they (1) had a clinical diagnosis of first‐ever unilateral thalamic stroke confirmed by experienced neurologists and MRI examinations; (2) in the subacute period (7 days to 1 month); (3) completed gait analysis and a structural brain MRI examination; and (4) provided written informed consent. Control participants were included if they met the following criteria: (1) aged 18 years or older; (2) had no history of stroke, Parkinson's disease, or other neurological conditions, such as brain injury and brain surgery, that could have affected the motor functions of participants; and (3) informed consent form signed by the participant or their guardian.

### 2.2 CSVD MRI Markers

According to the standards for reporting vascular changes on neuroimaging (STRIVE) criteria proposed by Wardlaw et al. (Wardlaw et al. 2013). Lacunes were defined as round or ovoid lesions (> 3 mm and < 20 mm diameter) occurring in the basal ganglia, internal capsule, centrum semiovale, or brainstem, with cerebrospinal fluid signal intensity on T2 and fluid‐attenuated inversion recovery (FLAIR), generally with a hyperintense rim on FLAIR and no increased signal on DWI, and defined as single or multiple. (Wardlaw et al. 2013) Enlarged perivascular spaces (EPVSs) were defined as small (< 3 mm) punctate (if perpendicular) and linear (if longitudinal to the plane of scan) hyperintensities on T2 images in the basal ganglia or centrum semiovale. According to a validated semiquantitative scale of 0 to 4, (Doubal et al. 2010), EPVSs in the basal ganglia were categorized as moderate to severe (grades 2–4). Deep and periventricular white matter hyperintensities (WMH) were coded from 0 to 3 on the Fazekas scale (Wardlaw et al. 2013) and categorized as either (early) confluent deep (score 2 or 3) or irregular periventricular extending into the deep white matter (score 3). Cerebral microbleeds (CMBs) were defined as homogeneous rounded lesions (2–10 mm in diameter) of signal loss on SWI. A CSVD compound score (Staals et al. 2014) ranging from 0 to 4 was established, depending on the presence or absence (1 or 0) of each CSVD MRI marker.

### 2.3 Gait Assessment

The “ReadyGo” system (Mo et al. 2024, Chen et al. 2024) (Beijing CAS‐Ruiyi Information Technology Co., Ltd.; details at [https://www.cas‐ruiyi.com/pages/page‐go.html]) was employed for quantitative gait analysis. This device, approved by the National Medical Products Administration of China, integrates a high‐resolution RGB (red/green/blue) camera 1920 × 1080 @30fps) and a time‐of‐flight (ToF) sensor to capture three‐dimensional (3D) motion and joint information of the human body. By utilizing deep learning algorithms for precise skeletal point positioning, the system eliminates the need for participants to wear any additional sensors, enabling the precise detection of postures and movements in 3D space. The system can recognize and track up to 32 skeletal joints, including the head, trunk, upper and lower limbs, and facial landmarks (Mo et al. 2024), The system analyzes gait by extracting spatiotemporal features from the motion data in each video frame. These features are processed through a bidirectional long short‐term memory (BiLSTM) deep neural network algorithm, specifically designed to calculate comprehensive gait parameters, ensuring accurate quantification of both temporal and spatial gait characteristics. The environmental deployment diagram of the device is included in the supplemental materials.

All balance‐and‐coordination‐related tests were performed by the professional neurologists (LCJ and YC) after the demonstration, and all parameters were measured by a professional neurologist under fall protection. To assess coordination, various measurement tools were utilized, including the heel‐knee‐shin test, finger‐nose test, TUG test, and Romberg test. Balance evaluation was carried out using the 5‐time sit‐to‐stand (5STS) test, tandem test, and semi‐tandem test. Notably, the annotated actions were manually reviewed and corrected to enhance the accuracy of the gait indicator calculations.

### 2.4 Statistical Analysis

Skewed distributions were expressed as medians and interquartile ranges (IQR). Categorical variables are presented as frequencies and percentages. The paired Wilcoxon test was used for within‐group comparisons of continuous variables, the independent samples t‐test or non‐parametric test was used for between‐group variables, and the chi‐square test was used for categorical variables. Given the symmetry of normal gait (< 6% limb difference), left and right parameters in HC were averaged. (Patterson et al. 2008). We decided to average the left and right limb parameters of each HC as the individual values for each HC. All data were analyzed using SPSS (version 23; SPSS, Chicago, United States) and GraphPad Prism (version 9.3.0; GraphPad Software, San Diego, United States), and a two‐sided *p* < 0.05 was considered statistically significant. For the correlation analysis, we used MATLAB R2018a to compute the correlation coefficients between gait parameters and CSVD markers for both the TI and HC groups. The correlation coefficients for each group were then transformed using Fisher's z‐transformation. We calculated the standardized difference in correlation values (Z‐diffuse) between the two groups and performed a Fisher z‐test to compute the p‐value and determine whether the differences were statistically significant. A p‐value less than 0.05 was considered statistically significant, indicating a meaningful difference between the two groups.

### 2.5 Ethical Statement

This study was approved by the Biomedical Ethics Review Committee of West China Hospital, Sichuan University (approval number 2022 (1200)). Written informed consent was obtained from all participants before the enrollment of this study

## 3 Result

### 3.1 Baseline Characteristics

**FIGURE 1:**
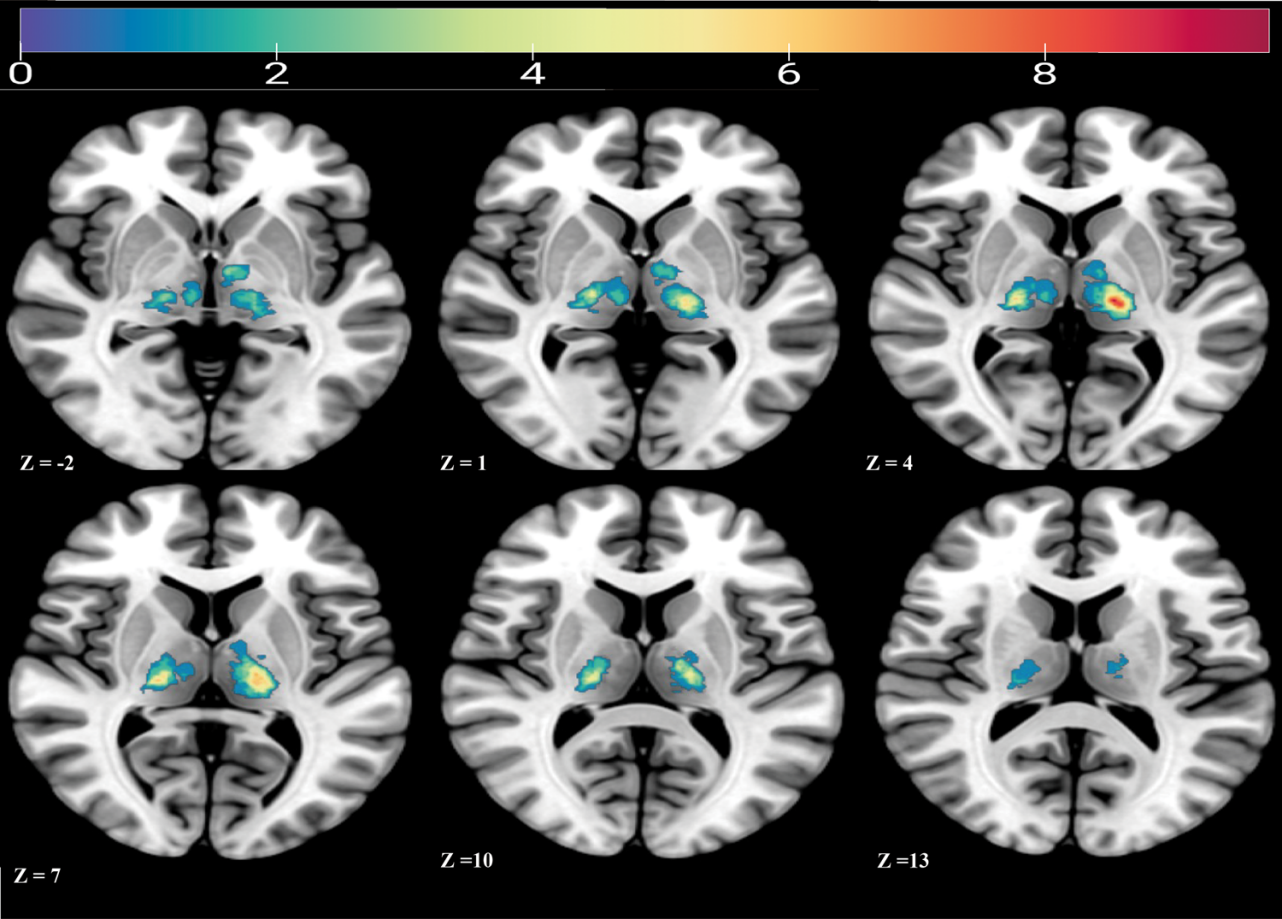
Overlap map of the infarction regions in TI patients.

In this study, we recruited a total of 90 participants, consisting of 45 individuals diagnosed with TI and an additional 45 individuals designated as HC. The baseline characteristics of all participants and the NIHSS and mRS Scores of patients have been consolidated and presented in Table 1. The NIHSS scores of all participants were focused exclusively on sensory abnormalities. However, due to 4 TI and 4 HC participants not cooperating with MRI scanning, for the assessment of lesion size and CSVD, we only included 41 TI and 41 HC participants. The overlap map of the patients is shown in Figure 1.

**TABLE 1:** Characteristics of patients TI and HC (for the infarct volume and the following sections, only 41 TI patients and 41 HC participants were included).

Parameters	Total	TI	HC	P‐value
Number of patients	90	45	45	−
Age (years (range))	61.73 ±8.751	62.82 ±10.216	60.64 ±6.935	0.240
Gender (female/male)	32/58	20/25	12/33	0.075
Weight (kg)	63.41 ±11.835	65.06 ±12.732	61.76 ±10.754	0.188
Height (cm)	165 (156‐168)	165 (160‐170.5)	162 (155‐168)	0.087
Duration of education (year)	9 (9‐14)	9(9‐14)	9 (9‐14)	0.909
Days post‐stroke	−	13 (8.0‐14.5)	−	−
Lesion laterality (left, %)	−	44%	−	−
NIHSS	−	1(1‐2)	−	−
mRS	−	1(1‐1)	−	−
Infarct volume(cm^3^)	−	0.21(0.075‐0.505)	−	−
Lacune	0(0‐0)	0(0‐1)	0(0‐0)	0.003
PWMH	1(1‐2)	1(1‐2)	1(0.5‐1)	**<** 0.001
DWMH	1(0‐1)	1(0‐2)	1(0‐1)	0.002
BG‐EPVS	1(1‐2)	1(1‐2)	1(1‐1)	0.002
CS‐EPVS	2(1‐2)	2(1‐2)	2(1‐2)	0.057
CMB	0(0‐1)	0(0‐1)	0(0‐0)	0.005
Total burden	1(0‐1.25)	1(0‐2)	0(0‐1)	**<** 0.001

### 3.2 Gait Parameter Analysis

We extracted 41 gait parameters for basic gait analysis. The analysis of gait parameters on the affected and healthy sideS of TI patients is shown in **Table** **2**. There were significant differences in variability(*p* = 0.045), average stride length (*p* = 0.042), maximum stride length (*p* = 0.017), average stride speed (*p* = 0.014), median stride speed (*p* = 0.039), and average swing velocity (*p* = 0.03) between the affected and healthy limbs in TI patients. (Supplemental Figure 1).

**TABLE 2:** Characteristics of gait parameters on TI patients in the unaffected side and affected side, and HC.

Parameters	TI			HC			
	Affected side. median (25%–75%)	Healthy side median (25%–75%)	P‐value	Average median (25%–75%)	P‐value affected	P value healthy	
Variation	9.096(7.637‐10.935)	8.41(6.29‐10.33)	0.045	8.494(6.835‐10.43)	0.264	0.684	
Stride Length	Average	1.065(0.997‐1.154)	1.1(1.013‐1.176)	0.042	1.153(1.094‐1.287)	0.001	0.009
	Median	1.103(1.02‐1.171)	1.106(1.022‐1.181)	0.312	1.167(1.109‐1.284)	<0.001	0.006
	Maximum	1.218(1.138‐1.324)	1.285(1.153‐1.391)	0.017	1.361(1.235‐1.506)	0.001	0.011
	Minimum	0.918(0.72‐0.994)	0.931(0.771‐1.018)	0.15	0.945(0.867‐1.088)	0.052	0.221
Stride Speed	Average	1.115(0.967‐1.224)	1.194(0.963‐1.26)	0.014	1.287(1.146‐1.4)	<0.001	0.001
	Median	1.117(0.984‐1.239)	1.170(0.982‐1.287)	0.039	1.285(1.144‐1.426)	<0.001	0.001
	Maximum	1.350(1.133‐1.527)	1.376(1.188‐1.54)	0.234	1.537(1.36‐1.717)	<0.001	<0.001
	Minimum	0.834(0.691‐1.049)	0.957(0.72‐1.072)	0.059	0.999(0.88‐1.147)	0.001	0.06
Swing velocity	Average	2.488(2.172‐2.806)	2.571(2.214‐2.889)	0.03	2.772(2.5‐2.991)	0.006	0.042
	Median	2.557(2.231‐2.867)	2.610(2.192‐2.889)	0.16	2.796(2.574‐3.052)	0.013	0.02
	Maximum	3.354(2.965‐3.659)	3.359(2.932‐3.684)	0.189	3.761(3.414‐4.038)	< 0.001	< 0.001
	Minimum	1.623(1.155‐2.03)	1.711(1.212‐2.176)	0.138	1.680(1.393‐2.222)	0.254	0.812
Stride frequency	Average	126.351(113.084‐135.383)	123.671(116.311‐134.21)	0.765	131.881(122.741‐141.332)	0.096	0.042
	Median	124.286(112.5‐133.516)	128.571(120‐128.571)	0.323	129.231(122.411‐138.668)	0.086	0.033
	Maximum	150(128.571‐163.636)	138.462(128.571‐163.636)	0.626	156.818(144.231‐168.409)	0.156	0.039
	Minimum	105.882(94.737‐112.5)	105.882(94.737‐112.5)	0.729	109.191(100.155‐116.25)	0.024	0.08
Standing phase	Average	67.197(65.849‐68.215)	66.836(65.646‐68.044)	0.394	66.101(65.017‐67.596)	0.081	0.092
	Median	67.204(65.889‐67.893)	66.667(65.858‐68.019)	0.626	66.059(65.198‐67.573)	0.032	0.05
	Maximum	71.875(70.27‐74.074)	72.414(70.185‐73.802)	0.608	71.388(69.798‐73.61)	0.214	0.335
	Minimum	61.765(59.688‐64.143)	62.5(60.357‐64)	0.345	60.315(58.23‐63.717)	0.232	0.125
Swing phase	Average	32.803(31.87‐34.277)	33.164(31.956‐34.353)	0.394	33.899(32.404‐34.983)	0.081	0.092
	Median	32.796(32.107‐34.111)	33.333(31.981‐34.142)	0.626	33.941(32.427‐34.802)	0.032	0.05
	Maximum	38.235(36‐40.825)	37.5(36‐39.643)	0.339	39.685(36.283‐41.77)	0.231	0.122
	Minimum	28.125(25.926‐29.73)	27.586(25.926‐29.630)	0.744	28.612(26.39‐30.202)	0.173	0.195
Step height	Average	0.120(0.099‐0.135)	0.117(0.102‐0.139)	0.524	0.124(0.106‐0.136)	0.548	0.455
	Median	0.120(0.097‐0.133)	0.114(0.0998‐0.134)	0.906	0.122(0.105‐0.137)	0.614	0.399
	Maximum	0.171(0.155‐0.196)	0.172(0.155‐0.194)	0.453	0.188(0.165‐0.204)	0.083	0.039
	Minimum	0.054(0.037‐0.081)	0.061(0.045‐0.0788)	0.045	0.063(0.045‐0.076)	0.631	0.926

Parameters	TI median (25%‐75%)	HC median (25%‐75%)	P‐value				
Walking speed	1.122(0.957‐1.197)	1.197(1.087‐1.295)	0.003				
Coordination	0(‐4.605‐0.000)	0(‐6.061‐0.000)	0.126				
Turn time	1.433(1.117‐1.617)	1.133(0.984‐1.333)	0.001				
Step width—average	0.137(0.128‐0.155)	0.126(0.117‐0.137)	0.001				
Step width—median	0.136(0.127‐0.153)	0.125(0.115‐0.138)	< 0.001				
Step width—Maximum	0.151(0.139‐0.172)	0.126(0.117‐0.137)	< 0.001				
Step width—minimum	0.119(0.112‐0.144)	0.125(0.115‐0.138)	0.569				
Double support phase—average	34.820(32.908‐35.654)	33.059(30.223‐34.978)	0.007				
Double support phase ‐median	34.483(33.333‐35.714)	33.333(31.665‐34.657)	0.009				
Double support phase—maximum	40.91(39.643‐42.641)	39.394(37.5‐42.308)	0.012				
Double support phase—minimum	26.923(23.905‐30.769)	24(20.711‐29.521)	0.026				

In addition, we compared the healthy side and affected side of TI patients with HC. Supplemental Table 1 shows that there were differences in 30 time‐and space‐related gait parameters between the two groups. The top 16 distinctive features (*p* < 0.001) are presented in Supplemental Figure 2 including stride length, average‐affected side, stride length maximum‐affected side, stride length—median—affected side, stride speed ‐average ‐affected side, stride speed average—healthy side, stride speed—median—affected side, stride speed—median—healthy side, stride speed—maximum‐affected side, stride speed—maximum—healthy side, stride speed—minimum‐affected side, swing velocity—maximum—affected side, swing velocity—maximum—healthy side, turn time, step width—average, step width‐median and step width ‐maximum.

### 3.3 Coordination Analysis

**FIGURE 2:**
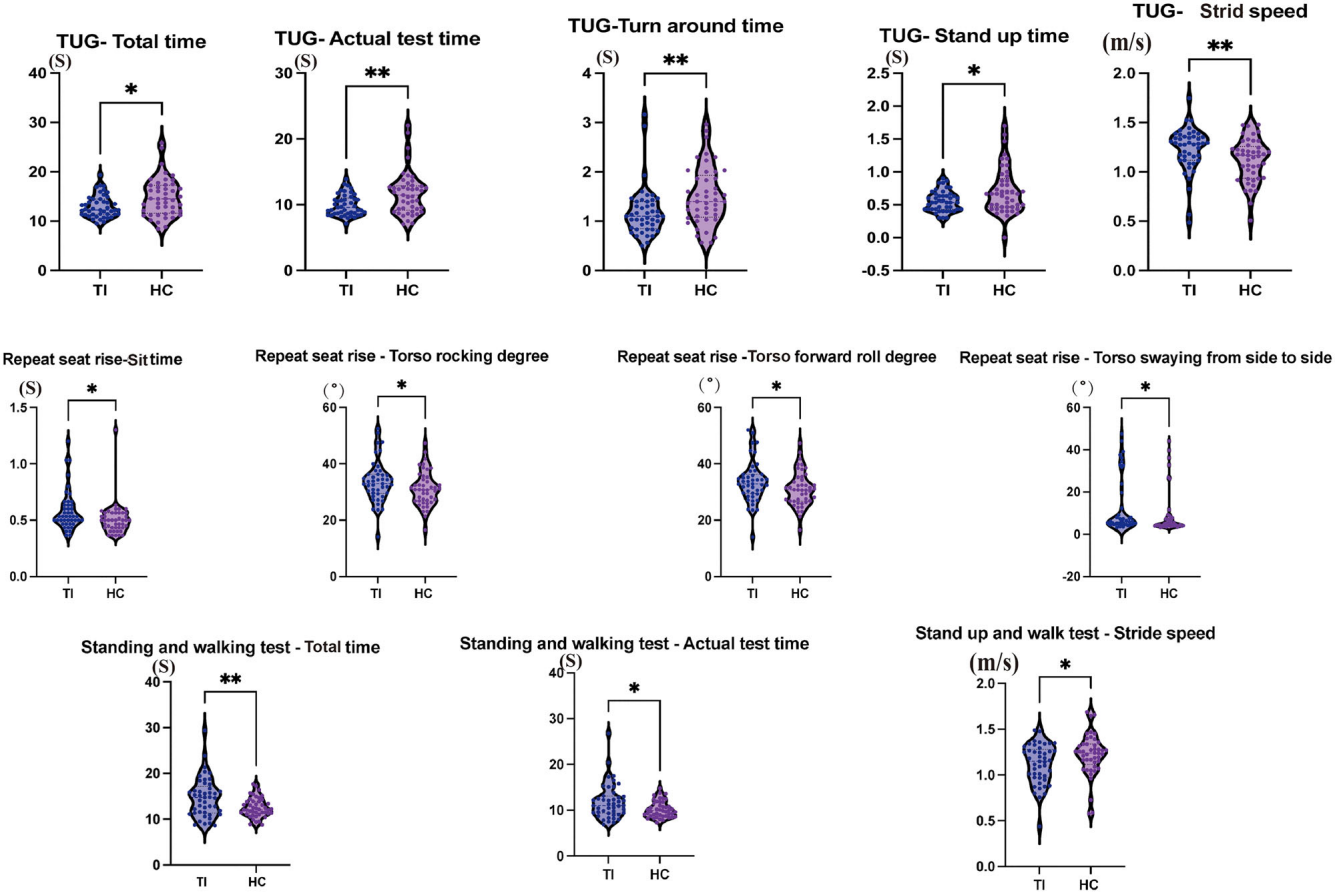
Figure 2A shows the distinctive coordination features (*p* < 0.05) between the TI and HC groups. The nonparametric test results of coordination tests between HC and TI are shown in the figure with significant differences (*p* < 0.05). the differences mainly focus on the TUG test. Figure 2B shows the distinctive Balance features (*p* < 0.05) between the TI and HC groups. The nonparametric test results of coordination tests between HC and TI are shown in the figure with significant differences (p < 0.05).

We evaluated the participants' coordination using the TUG and Romberg's test, and the results are displayed in Supplemental Table 1. There were no statistically significant differences between the two groups in Romberg's test, but variations were observed in total time, actual test time, turn time, stand up time, and stride speed during the TUG test (Figure 2A).

### 3.4 Balance Analysis

We employed the 5STS test, the tandem test, and the semi‐tandem test to assess the participants' balance. The results are presented in Supplemental Table 2. Differences were observed between the TI and HC groups in sit time, torso rocking degree, torso forward roll degree, and torso swaying from side to side during the 5STS test. Additionally, differences were found in the total time, actual time, and walking speed during the TUG test. (Figure 2B)

### 3.5 Correlation Analysis Between Gait and CSVD Markers

**FIGURE 3:**
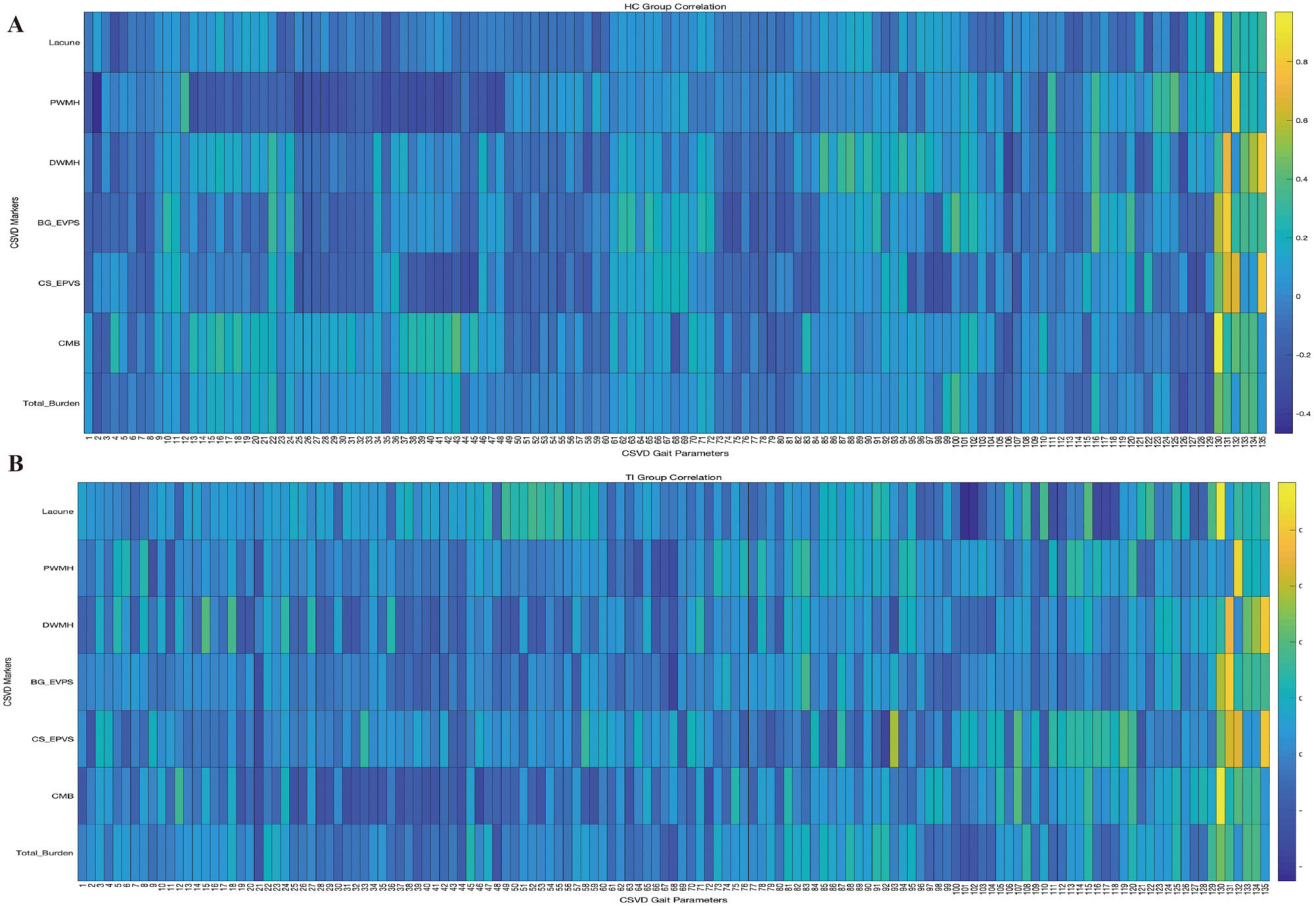
Figure 3A shows the correlation coefficients between gait parameters and CSVD in TI patients, while Figure 3B displays the correlation coefficients between gait parameters and CSVD in HC. The names of the gait parameters from 1 to 135 are presented in the Supplementary materials.

Figure 3 shows the correlation coefficients between gait parameters and CSVD in TI and HC. The names of the gait parameters from 1 to 135 are presented in the Supplementary materials. Additionally, Table 3 presents the parameters with a *p*‐value < 0.05 for the correlation tests between HC and TI. Among these, the differences in step width—max and tandem stance—trunk sway degree with total burden are statistically significant.

**TABLE 3:** Table 3 presents all the indicators with significant differences (*p* < 0.05) in the correlation difference analysis between gait and CSVD markers in TI patients and HC, along with their correlation values (r).Δ represents the value of the affected side minus the value of the healthy side.

		TI‐correlation	HC‐correlation	P‐value
LACUNES	Step frequency‐max ‐affected	0.340	−0.115	0.050
	Step frequency‐min ‐healthy	0.219	−0.112	0.042
	Step width‐min	−0.453	0.049	0.019
	Repeated chair rise‐stand up time	0.391	−0.042	0.047
	TUG‐ total time	0.359	−0.250	0.006
	TUG‐stand up time	0.294	−0.271	0.011
	Tandem stance‐trunk sway degree	0.378	−0.153	0.016
DWMH	Step height‐max‐Δ	−0.277	0.194	0.036
	Eyes closed stance—total time	0.055	−0.375	0.050
BG‐EPVS	Stance phase—max—healthy	−0.351	0.169	0.014
	Swing phase—average—healthy	0.191	−0.286	0.039
CS‐EPVS	Swing phase‐min‐healthy	0.347	−0.169	0.015
	Step width‐max	−0.139	0.391	0.016
	Repeated chair rise—sit down speed	−0.030	0.414	0.040
	Step frequency—min—affected	0.317	−0.118	0.015
	Stance phase‐Max‐Healthy	−0.230	0.217	0.048
	Swing phase‐min‐Δ	0.233	−0.217	0.046
	Step height‐max‐ healthy	−0.357	0.103	0.012
	Step height‐ max‐Δ	0.549	0.103	0.025
	TUG—total time	0.402	−0.248	0.003
**CMB**	Stride length‐max‐Δ	−0.287	0.195	0.032
	Step speed‐max‐affected	−0.291	0.088	0.032
	Swing speed—average—affected	−0.259	0.246	0.036
	Swing speed—average—healthy	−0.186	0.246	0.045
	Swing speed‐median—affected	−0.253	0.271	0.025
	Swing speed—median—healthy	−0.240	0.271	0.036
	Swing speed‐max‐affected	−0.166	0.271	0.009
	Stance phase‐max‐Δ	−0.318	0.157	0.033
	TUG—total time	0.391	−0.069	0.035
	Repeated chair rise—stand up time	0.308	−0.183	0.028
	Tandem stance‐ trunk sway degree	0.338	−0.290	0.045
Total_Burden	Step width—max‐	−0.147	0.343	0.028
	Tandem stance‐ trunk sway degree	0.338	−0.270	0.006

## 4 Discussion

This study aimed to examine whether there are differences in gait, balance, and coordination among TI patients. In contrast to previous research, this study represents the pioneering baseline investigation focused on detailed and quantitative gait analysis in TI patients. (Sharma et al. 2023; Kurosu et al. 2011) Its primary objective is to explore the specific gait patterns observed in patients with thalamic infarction and to provide valuable insights for guiding subsequent personalized rehabilitation efforts. The results revealed that even in TI patients without motor impairments, discernible distinctions persisted in terms of meticulous motor parameters, balance, and coordination.

The analysis of gait parameters revealed significant changes in the walking pattern of patients with TI. When comparing the stride length between the affected and unaffected sides of TI patients, we observed an impact on the symmetry of stride length, with the affected side having a smaller stride length compared with the unaffected side. Furthermore, compared with HC, both the affected and unaffected sides of TI patients exhibited reduced stride length. This reduction may be attributed to TI patients choosing to shorten their stride length to enhance balance due to decreased propulsion on the affected side caused by hemiplegia (Roelker et al. 2019). This is consistent with previous findings in CSVD research, where white matter lesions (WMLs) and lacunar infarctions in the thalamus have been associated with reduced stride length (de Laat et al. 2012; Su et al. 2022). Karlijn F. further suggested that thalamic lacunes may also disrupt gait rhythm, subsequently leading to a decline in walking speed. (de Laat et al. 2010). Furthermore, the affected side exhibits a noticeably higher variation, indicating an unstable and inconsistent stride length. On the healthy side, there is a significant decrease in stride frequency, suggesting a slower rhythm of walking. These observations highlight the fact that even when sacrificing stride length, TI patients still experience an unstable and irregular gait pattern. As a result, they require increased attention and effort to maintain balance. Additionally, in terms of spatial parameters, there was a significant increase in step width among TI patients. The magnitude of step width change in stroke patients depends on the type and severity of the stroke as well as patient rehabilitation. Increasing step width serves as a compensatory mechanism to enhance stability and prevent falls. However, excessively wide step widths can result in inefficient walking and abnormal gait patterns. (Koch et al. 2019; Reimold et al. 2021) Overall, our study, using precise measurements, revealed spatial asymmetry in TI patients, characterized by reduced step length and increased step width.

In terms of temporal parameters, TI patients exhibit specific changes. The stance time on the affected side increases, while the swing time decreases, and the double support phase time increases. These alterations indicate an impaired ability to prepare the body for forward progression and require more time for the center of gravity to shift. This temporal asymmetry, often characterized by an extended swing time and/or prolonged standing time on the affected side compared to the unaffected limb, is frequently observed in hemiplegic gait. Our findings support the presence of temporal asymmetry in the gait of TI patients. Speed serves as an overall indicator of gait performance and is closely related to various other spatial‐temporal parameters. Our results demonstrate that TI patients exhibit reduced stride speed, reflecting a slower walking pace. Slower speed can be attributed to factors such as limited movement recovery, impaired balance, reduced muscle strength, and a significant negative impact on an individual's level of independence. (Viosca et al. 2005). Koblinsky et al. observed that gait speed is directly associated with thalamic cerebral blood flow (CBF) in older adults (Koblinsky et al. 2020) and recent studies have further suggested that gait velocity is strongly correlated with thalamic volume in patients with subcortical vascular cognitive impairment (SVCI) (Zhou et al. 2024). These findings are consistent with our study, The thalamus serves as a critical neural substrate influencing gait velocity in patients. Previous studies have demonstrated that gait asymmetry is commonly observed in individuals with stroke. (Balaban and Tok 2014, Patterson et al. 2010) These studies have highlighted the presence of temporal asymmetry in different gait parameters, including stance time, single stance time, double support time, and swing time. Our study reinforces these findings and provides additional evidence that even in the absence of significant motor impairments, patients with TI exhibit a characteristic post‐stroke gait pattern (Sheffler and Chae 2015).

Meanwhile, as one of the most used scales for post‐stroke gait assessment in clinical (Cai et al. 2021), we utilized the Tinetti scale to evaluate our TI patients (Supplemental Table 3). We found that although there were variations in the final scores, both the TI and HC groups scored above 24 points, indicating a normal range of performance for both groups. However, after assessing the coordination and balance of TI patients, our results found that TI patients require more time and effort to perform actions such as turning, standing up, fine motor tasks (e.g., finger‐to‐nose test, heel‐knee‐shin test), and maintaining body balance. Although these changes do not significantly affect their daily life, they still exhibit typical post‐stroke gait patterns. Despite investigating hemiplegic gait during the development of gait analysis and rehabilitation methods, the causal relationship between stroke‐induced impairments and post‐stroke gait patterns remains not fully understood (Koch et al. 2019). Therefore, we included markers of CSVD in the study and conducted a preliminary correlation analysis between gait and CSVD. Previous studies have shown that CSVD is associated with a slower step speed, shorter stride length, longer stance time, prolonged swing time, increased step width, and greater gait asymmetry. (Blumen et al. 2023, Chen et al. 2024, Ma et al. 2022, Jiang et al. 2023) This may be because thalamic infarction potentially disrupts the cortico‐basal ganglia‐thalamic loop, leading to an increased CSVD burden, which directly exacerbates gait disturbances. This makes the cumulative effects of CSVD more pronounced in TI patients. It is also possible that the cognitive impairments caused by CSVD indirectly exacerbate gait disturbances. It is worth noticing that cognitive (Cai et al. 2021) and sensory impairments are more commonly observed in TI patients, rather than motor impairments. Previous studies have highlighted a close relationship between cognitive impairment and gait disturbances (Mo et al. 2024, Chen et al. 2024), which may be attributed to the shared neural regions involved in both functions. Andrea L (Rácz et al. 2018) suggested that the link between slowed gait and cognitive impairment is supported by a shared neural substrate, including a reduced right hippocampus. As for the HC group, healthy elderly individuals may partially offset the negative effects of CSVD through preserved cognitive reserves or adjustments in motor strategies.

Based on the findings of our study, rehabilitation strategies for TI patients should include personalized gait training to address spatial asymmetry, focusing on step length and step width correction. Balance and coordination exercises are essential, with an emphasis on dynamic stability and fine motor control. Given the close relationship between cognition and gait, incorporating dual‐task training and cognitive rehabilitation may further enhance recovery. Additionally, rhythm‐guided training and sensory feedback modulation can optimize gait stability and efficiency. (Maclean et al. 2014; Di Russo et al. 2021)

To our knowledge, this is the first study to objectively evaluate the gait patterns of TI patients using quantifiable data. Several methodological issues and limitations should be considered. First, the sample size in this study was relatively small, and no significant differences were observed in gait parameters. Additionally, this is a cross‐sectional study. We plan to expand the sample size and conduct longitudinal cohort studies to explore the causal relationship between infarction and CSVD. While the controlled study environment was appropriate, it cannot replicate the complexity of real‐world conditions, where obstacles, slopes, and surface variations may further impact gait patterns. Therefore, the gait assessment here may not capture all the challenges faced in daily life. Furthermore, future studies should incorporate neuroimaging techniques such as DTI and fMRI to explore the pathophysiological links between cognitive impairments and gait changes. Integrating neural imaging data with gait analysis will be crucial for understanding the underlying mechanisms of these associations.

## 5 Conclusion

We conducted a quantitative analysis of spatiotemporal gait data in TI patients by utilizing advanced gait analysis instruments, our study discovered that even in TI patients who were considered to have no motor impairments clinically, there were typical alterations in post‐stroke gait spatiotemporal parameters. Additionally, noticeable deficits in coordination and balance were observed. Future research endeavors should aim to elucidate the pathological processes underlying gait disturbances in more detail and facilitate individualized therapies.

## Author Contributions

**Congjun Li**: Conceptualization, investigation, writing ‐ original draft, writing ‐ review and editing, software, formal analysis, methodology, data curation. **Chen Ye**: Formal analysis, data curation, validation, investigation, writing ‐ original draft. **Sun Rui**: data curation, software, formal analysis. **Ruosu Pan**: Software, validation, methodology. **Shuai Jiang**: Validation, investigation. **Yuyign Yan**: Visualization, formal analysis. **Tang Yang**: Formal analysis, data curation. **Le Cao**: Data curation, formal analysis. **Hang Wang**: Data curation, formal analysis. **Youjie Wang**: Methodology. **Junfeng Liu**: Validation. **Wendan Tao**: Conceptualization. **Bo Wu**: Writing ‐ original draft, writing ‐ review and editing, funding acquisition, project administration, visualization, resources, conceptualization.

## Conflicts of Interest

The authors declare that the research was conducted in the absence of any commercial or financial relationships that could be construed as a potential conflict of interest.

### Peer Review

The peer review history for this article is available at https://publons.com/publon/10.1002/brb3.70582

## Supporting information



Supporting Information

## Data Availability

Upon a reasonable request to the corresponding author, data supporting the study's results can be provided to qualified investigators.
