# Supplementary material for: Discrepant Spatiotemporal Characteristics of Gait Impairments in Thalamic Infarction Patients
Source: Brain Behav. 2025 May 26;15(5):e70582. doi: 10.1002/brb3.70582 (PMC12105651; doi:10.1002/brb3.70582)
Supplement: Supplementary file 1 — Supporting Information [file BRB3-15-e70582-s001.docx]

**Supplemental Materials**

**Supplemental method**

The following supplementary image shows our equipment placement diagram. We have placed prominent markers on the floor to indicate where the subjects should take action, ensuring consistency in gait measurements


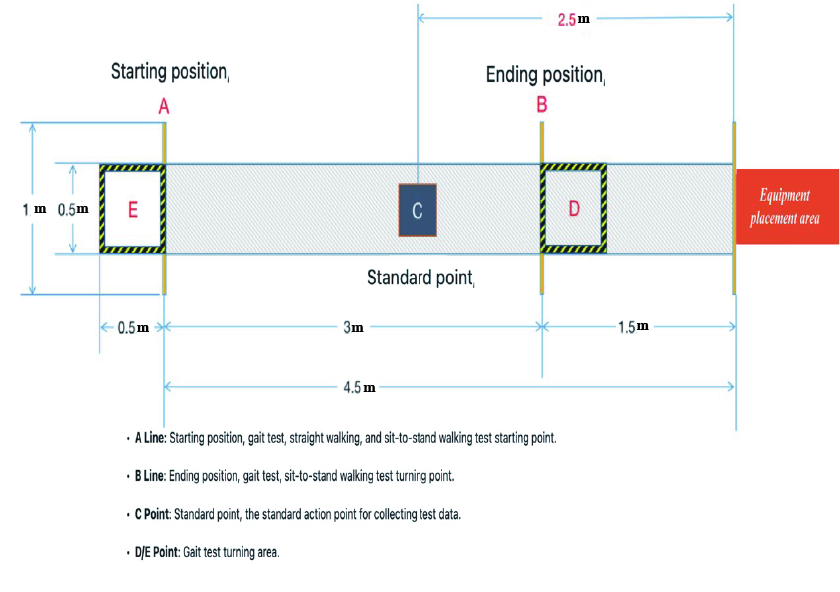


The participants were then instructed to walk at their regular speed to a marker placed 3 m away, turn around, and walk back. After the task, the video was reviewed to ensure that the software recognized the key actions and body markers properly. The stance phase (left and right), swing phase (left and right), double-stance phase (left and right), stride length (left and right), speed, step width, step height (left and right), stride velocity (left and right), and swing velocity (left and right) were also recorded.

The Standing with Eyes Closed Test, Timed Up and Go Test (TUG), 5 Times Repeat Seat Rise, Tandem Standing, Semi-Tandem Standing, and the balance test in the Tinetti Test were performed at point C, following a demonstration by LCJ and YC

1 Coordination

Left foot to right foot length ratio.

2. Walking speed (m/s) :

Distance/time between the start point of the walking cycle and the endpoint of the walking cycle (excluding the turn cycle)

3. Stride length (m) :

Left stride: The distance between two landing of the left foot.

Right stride: The distance between two landing of the right foot

4. Step height (m):

Left step height: the highest distance from the ground during the swing of the left foot.

Right step height: the highest distance from the ground during the swing of the right foot

5, Step frequency

The number of steps taken per unit of time, typically measured in steps per minute (SPM)

6. Step width (m) :

Foot width

7, Stride speed (m/s) :

Left stride speed: left stride length/cycle time per left stride length.

Right stride speed: right stride length/cycle time per right stride

8, Swing speed (m/s) :

Left foot swing speed: left foot swing distance/left foot swing time.

Right foot swing speed: right foot swing distance/right foot swing time.

9, Swing phase (%) :

Left swing phase: left foot swing time/left stride time.

Right swing phase: right foot swing time/right stride time

10, Standing phase (%) :

Left standing phase: left foot standing time/left stride time.

Right standing phase: right foot standing time/right stride time

11, Double support phase (%):

The proportion of time spent standing on both feet during each stride cycle.

12. Variation

Variance left standard deviation of left foot stride and standard deviation of left foot stride.

Variance right: standard deviation of right foot stride and standard deviation of right foot stride

1. TUG

1. Total time (s):

Time to complete the entire test.

2, turn time (s):

The time it takes to turn during a gait.

3, stand up time (s):

Time to get up from the seat at the start of the test.

4. Sitting time (s):

The time from standing up to sitting down at the end of the test.

5, standing speed (s):

How fast you get up from your seat.

6. Sitting speed (s):

From standing up to sitting down

Torso wobble

The degree of shaking is comprehensive, before and after four directions

**Movement cycle definition:** A complete gait cycle was defined as the sequence from the initial contact of one foot to the subsequent initial contact of the same foot. Key gait phases, including the stance phase (left and right), swing phase (left and right), and double stance phase (left and right), were extracted from the 3D motion data using the BiLSTM algorithm. Other parameters, such as stride length, stride velocity, and step width, were calculated based on joint trajectories and time-series data, ensuring consistency and reproducibility across all measurements.

**High-resolution 3D T1-weighted structural MRI：**Participants underwent scanning on a 3T MRI scanner (MAGNETOM Prisma; Siemens 2 Healthineers, Erlangen, Germany) with a 32-channel head coil. HR-sMRI sequence parameters:A 3D-T1 BRAVO sequence (Axial MRI 3D T1-weighted Brain Volume Sequence) was employed with the following parameters: repetition time (TR) = 7.2 ms, echo time (TE) = 3.0 ms, isotropic voxel size = 1×1×1 mm³, effective field of view (FOV) = 256×256 mm², matrix size = 256×256 mm², number of slices = 152, no interslice gap, flip angle (FA) = 12°, and a scan duration of approximately 3 minutes and 43 seconds.

**Lesion volume：**Using the Clinical Toolbox (https://nitrc.org/projects/clinicaltbx/) integrated into the SPM12 software (Statistical Parametric Mapping Package, https://www.fil.ion.ucl.ac.uk/spm/), based on MATLAB R2018b (The MathWorks Inc., Natick, Massachusetts, US), individual lesion masks and T1WI-BRAVO images were registered to the Montreal Neurological Institute (MNI) standardized space with an isotropic voxel size of 1×1×1 mm³. After standardization, the lesion volume for each patient (in cm³) was obtained based on the standardized lesion VOI files.

**Supplemental figure 1|The distinctive gait features (P < 0.05) in the TI group. The paired calculation results of gait parameters on the healthy side and the affected side of TI are shown in the figure with significant differences (p<0.05)**

**
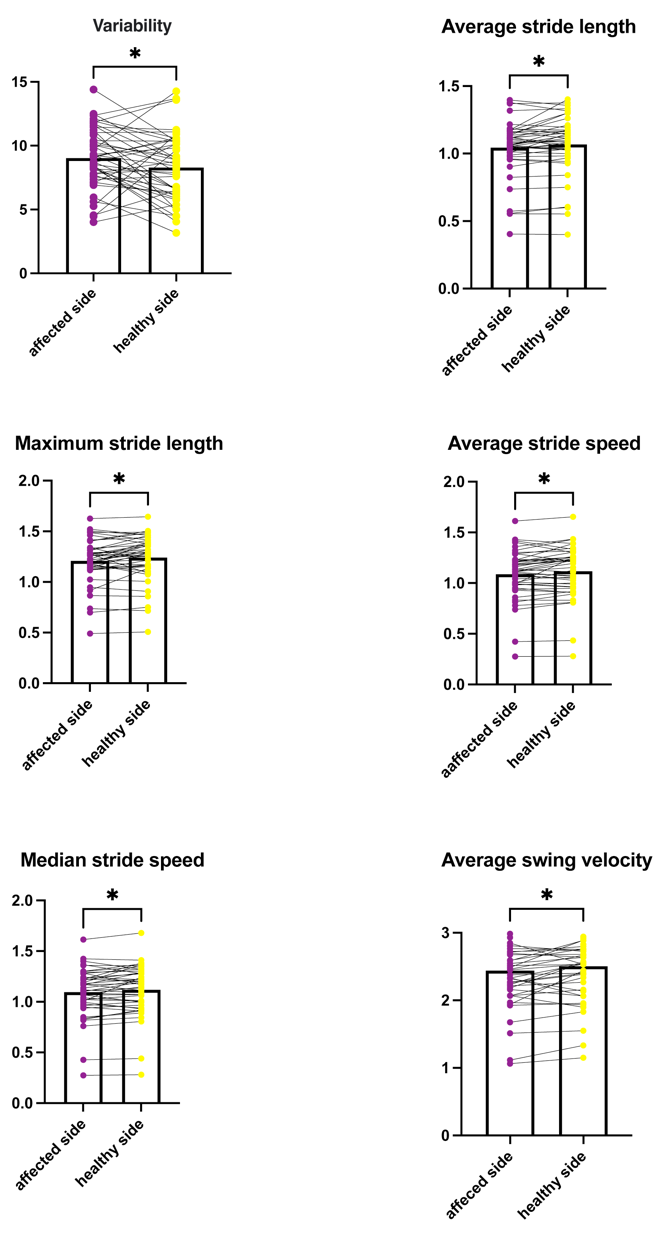
**

**Supplemental figure 2| The top 16 distinctive gait features (P < 0.001) between the TI and HC groups.**

**
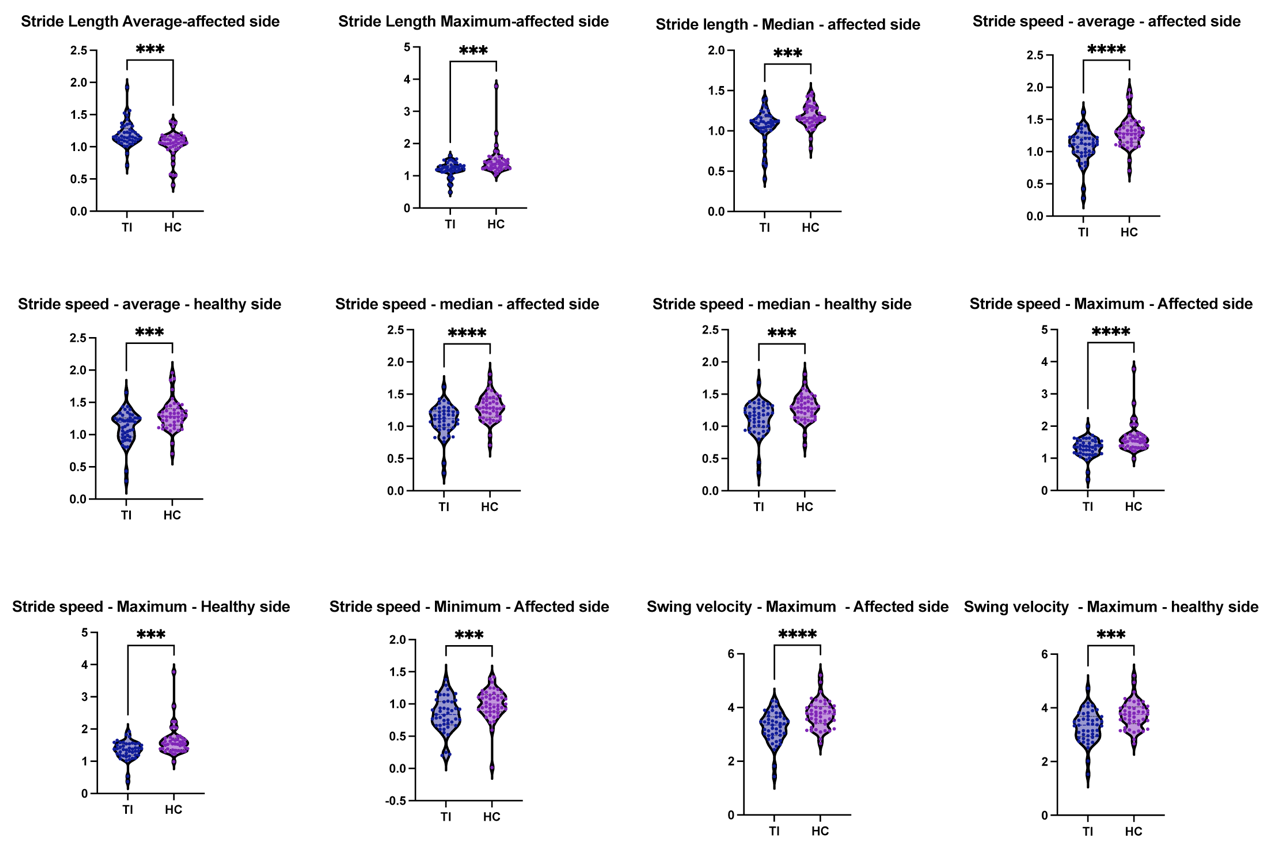
**

**Supplemental Table**

**Supplemental table 1| Characteristics of the coordination parameters in TI and HC**

|  | | Parameters | | TI  Mean (25%-75%) | | HC  Mean (25%-75%) | | | P value |  |  |
| --- | --- | --- | --- | --- | --- | --- | --- | --- | --- | --- | --- |
| Standing With Eyes Closed Test | | Torso Swaying Degree | | 10.731(7.059-13.969) | | 10.19(7.0975-14.235) | | | 0.994 |  |  |
|  |  | Torso Leaning Forward and Swaying | | 10.612(6.534-14.235) | | 10.77(6.534-14.23) | | | 0.965 |  |  |
|  |  | Torso Swaying from Side to Side | | 3.942(2.892-6.608) | | 4.185(2.658-4.668) | | | 0.417 |  |  |
|  |  | Total Time | | 11.464 (11.241-11.66) | | 11.76 (11.30-11.89) | | | 0.19 |  |  |
| Timed Up and Go Test (TUG ) | | Total Time | | 14.5(11.615-17.3) | | 12.98 (11.34-14.49) | | | **0.022** |  |  |
|  |  | Actual Test Time | | 11.47(9.168-12.865) | | 9.933(8.655-11.02) | | | **0.004** |  |  |
|  |  | Turn Time | | 1.4 (1.083-1.933) | | 1.190(0.850-1.4) | | | **0.002** |  |  |
|  |  | Stand Up Time | | 0.666(0.466-0.9) | | 0.5538(0.433-0.6495) | | | **0.027** |  |  |
|  |  | Sitting Time | | 0.6(0.5-0.783) | | 0.5759 (0.483-0.666) | | | 0.115 |  |  |
|  |  | Walking Speed | | 1.167 (0.937-1.257) | | 1.228(1.117-1.358) | | | **0.01** |  |  |
|  | |  | |  | |  | | |  |  |  |
| Coordination test | | Heel knee shin test | | Affected side | | 0.333 | | 0.022 | **0.009** | | |
|  |  |  |  | Healthy side | | 0.311 | | 0.111 | **0.001** | | |
|  |  | Finger-nose test | | Affected side | | 0.244 | | 0.089 | **0.003** | | |
|  |  |  |  | Healthy side | | 0.178 | | 0 | **0.049** | | |
|  |  | Stand with eyes closed | | | | 2 | | 2 | 1 | | |
|  |  | Total | | | | 3.044 | | 2.222 | **<0.001** | | |

**Supplemental table 2|** **Characteristics of the balance parameters in TI and HC**

| Parameters | | TI  Median (25%-75%) | HC  Median (25%-75%) | P value |
| --- | --- | --- | --- | --- |
| 5’Times  Repeat Seat Rise | Total Time | 15.93 (13.58-21.14) | 15.15 (14.09-19.10) | 0.291 |
|  | Stand Up Speed | 0.456(0.406-0.531) | 0.4702(0.4025-0.5375) | 0.453 |
|  | Stand Up Time | 1.030(0.742-1.283) | 0.91076(0.6915-1.066) | 0.207 |
|  | Sit Speed | 0.409(0.354-0.490) | 0.4495(0.3815-0.5055) | 0.05 |
|  | Sit Time | 0.583(0.483-0.633) | 0.506(0.433-0.566) | **0.011** |
|  | Seat Stay Time | 1.029(0.742-1.283) | 0.917(0.708-1.066) | 0.239 |
|  | Torso Rocking Degree | 33.71(29.468-36.237) | 31.1 (26.823-34.82) | **0.047** |
|  | Torso Forward Roll Degree | 33.641 (29.443-36.231) | 31.040 (26.760-34.716) | **0.048** |
|  | Torso Swaying from Side to Side | 12.719 (4.678-16.044) | 9.026 (4.016-7.495) | **0.029** |
| Tandem  Standing | Total Time | 9.29(10.0102002-10.06308315) | 10.237 (10.016-10.053) | 0.881 |
|  | Body Wobble | 13.508 (9.104-16.45) | 13.665 (8.686-16.964) | 0.796 |
|  | Torso Forward Rocking Degree | 13.26 (8.835-16.342) | 13.385 (8.610-16.093) | 0.843 |
|  | Torso Swaying from Side To Side | 6.473(3.483-8.746) | 5.397 (2.604-6.233) | 0.052 |
| Semi-Tandem Standing - | Total Time | 10.334(10.02-10.063 | 10.208 (10.0212-10.055) | 0.559 |
|  | Body Wobble | 11.106 (7.191-14.373) | 11.647(7.714-14.562) | 0.9 |
|  | Torso Forward Rocking Degree | 10.640 (6.104-14.359) | 11.303 (7.193-14.186) | 0.775 |
|  | Torso Swaying from Side To Side | 5.624 (2.906-5.973) | 4.72(2.966-5.516) | 0.377 |

**Supplemental table 3|** **Characteristics of the** **Tinetti test in TI and HC**

| Parameters | | | TI | | HC | P value |  |
| --- | --- | --- | --- | --- | --- | --- | --- |
| Tinetti  Test | Balance tests | Sitting balance | 1.956 |  | 2 | 0.317 |  |
|  |  | Arises | 1.844 |  | 2 | **0.006** |  |
|  |  | Attempts to arise | 0.933 |  | 0.978 | 0.309 |  |
|  |  | Immediate standing balance | 1.978 |  | 2 | 0.080 |  |
|  |  | Standing balance | 1.911 |  | 1.978 | 0.304 |  |
|  |  | Nudged | 1.889 |  | 2 | **0.042** |  |
|  |  | Eyes closed | 2 |  | 2 |  |  |
|  |  | Tuming 360° | 1.844 |  | 2 | 0.012 |  |
|  |  | Sitting down | 1.933 |  | 2 | 0.08 |  |
|  | Gait test | Initiation of gait | 0.956 |  | 1 |  |  |
|  |  | Step symmetry | 0.933 |  | 1 | 0.080 |  |
|  |  | Step continuity | 1 |  | 1 | 1 |  |
|  |  | Path | 1.733 |  | 1.911 | **0.028** |  |
|  |  | Trunk | 0.956 |  | 1 | 0.155 |  |
|  |  | Walking stance | 0.578 |  | 0.844 | **0.006** |  |
|  |  | Step length-affected | 1 |  | 1 | 1 |  |
|  |  | Step length-healthy | 1 |  | 1 | 1 |  |
|  |  | Step height-affected | 0.956 |  | 1 | 0.317 |  |
|  |  | Step height-healthy | 0.978 |  | 1 | 0.155 |  |
|  |  | Total | 26.067 |  | 27.711 | **<0.001** |  |

The X-axis labels for TI1-135 in Figure 3.

. 1 Step speed

2. Coordination

3. Variability affected

4. Variability health

5. affected-health difference

6. Swing phase affected

7. Swing phase health

8. affected-health difference

9. Stance phase affected

10. Stance phase health

11. affected-health difference

12. Turn time

13. Stride-mean-affected

14. Stride-mean-health

15. affected-health difference

16. Stride-median-affected

17. Stride-median-health

18. affected-health difference

19. Stride-max-affected

20. Stride-max-health

21. affected-health difference

22. Stride-min-affected

23. Stride-min-health

24. affected-health difference

25. Step speed-mean-affected

26. Step speed-mean-health

27. affected-health difference

28. Step speed-median-affected

29. Step speed-median-health

30. affected-health difference

31. Step speed-max-affected

32. Step speed-max-health

33. affected-health difference

34. Step speed-min-affected

35. Step speed-min-health

36. affected-health difference

37. Swing speed-mean-affected

38. Swing speed-mean-health

39. affected-health difference

40. Swing speed-median-affected

41. Swing speed-median-health

42. affected-health difference

43. Swing speed-max-affected

44. Swing speed-max-health

45. affected-health difference

46. Swing speed-min-affected

47. Swing speed-min-health

48. affected-health difference

49. Step frequency-mean-affected

50. Step frequency-mean-health

51. affected-health difference

52. Step frequency-median-affected

53. Step frequency-median-health

54. affected-health difference

55. Step frequency-max-affected

56. Step frequency-max-health

57. affected-health difference

58. Step frequency-min-affected

59. Step frequency-min-health

60. affected-health difference

61. Stance phase-mean-affected

62. Stance phase-mean-health

63. affected-health difference

64. Stance phase-median-affected

65. Stance phase-median-health

66. affected-health difference

67. Stance phase-max-affected

68. Stance phase-max-health

69. affected-health difference

70. Stance phase-min-affected

71. Stance phase-min-health

72. affected-health difference

73. Swing phase-mean-affected

74. Swing phase-mean-health

75. affected-health difference

76. Swing phase-median-affected

77. Swing phase-median-health

78. affected-health difference

79. Swing phase-max-affected

80. Swing phase-max-health

81. affected-health difference

82. Swing phase-min-affected

83. Swing phase-min-health

84. affected-health difference

85. Step height-mean-affected

86. Step height-mean-health

87. affected-health difference

88. Step height-median-affected

89. Step height-median-health

90. affected-health difference

91. Step height-max-affected

92. Step height-max-health

93. affected-health difference

94. Step height-min-affected

95. Step height-min-health

96. affected-health difference

97. Step width-mean-affected-health

98. Step width-median-affected-health

99. affected-health difference

100. Step width-max-affected-health

101. Step width-min-affected-health

102. affected-health difference

103. Eyes closed standing-trunk shake degree

104. Eyes closed standing-trunk forward tilt shake degree

105. Eyes closed standing-trunk lateral shake degree

106. Eyes closed standing-total time

107. Get-up walk test-total time

108. Get-up walk test-actual test time

109. Get-up walk test-turn time

110. Get-up walk test-stand time

111. Get-up walk test-sit time

112. Get-up walk test-step speed

113. Repeated seat stand-total time

114. Repeated seat stand-stand speed

115. Repeated seat stand-stand time

116. Repeated seat stand-sit speed

117. Repeated seat stand-sit time

118. Repeated seat stand-chair stay time

119. Repeated seat stand-trunk shake degree

120. Repeated seat stand-trunk forward tilt shake degree

121. Repeated seat stand-trunk lateral shake degree

122. Get-up walk test-total time

123. Get-up walk test-actual test time

124. Get-up walk test-turn time

125. Get-up walk test-stand time

126. Get-up walk test-sit time

127. Get-up walk test-step speed

128. Tandem standing-total time

129. Tandem standing-trunk shake degree

130. Tandem standing-trunk forward tilt shake degree

131. Tandem standing-trunk lateral shake degree

132. Half tandem standing-total time

133. Half tandem standing-trunk shake degree

134. Half tandem standing-trunk forward tilt shake degree

135. Half tandem standing-trunk lateral shake degree

The X-axis labels for HC 1-135 in Figure 3.

1. Step speed

2. Coordination

3. Variability Left-Right Average

4. Variability Left-Right Average

5. Left-Right Average

6. Swing Phase Left-Right Average

7. Swing Phase Left-Right Average

8. Left-Right Average

9. Stance Phase Left-Right Average

10. Stance Phase Left-Right Average

11. Left-Right Average

12. Turning Time

13. Step Length - Mean - Left-Right Average

14. Step Length - Mean - Left-Right Average

15. Left-Right Average

16. Step Length - Median - Left-Right Average

17. Step Length - Median - Left-Right Average

18. Left-Right Average

19. Step Length - Max - Left-Right Average

20. Step Length - Max - Left-Right Average

21. Left-Right Average

22. Step Length - Min - Left-Right Average

23. Step Length - Min - Left-Right Average

24. Left-Right Average

25. Stride Speed - Mean - Left-Right Average

26. Stride Speed - Mean - Left-Right Average

27. Left-Right Average

28. Stride Speed - Median - Left-Right Average

29. Stride Speed - Median - Left-Right Average

30. Left-Right Average

31. Stride Speed - Max - Left-Right Average

32. Stride Speed - Max - Left-Right Average

33. Left-Right Average

34. Stride Speed - Min - Left-Right Average

35. Stride Speed - Min - Left-Right Average

36. Left-Right Average

37. Swing Speed - Mean - Left-Right Average

38. Swing Speed - Mean - Left-Right Average

39. Left-Right Average

40. Swing Speed - Median - Left-Right Average

41. Swing Speed - Median - Left-Right Average

42. Left-Right Average

43. Swing Speed - Max - Left-Right Average

44. Swing Speed - Max - Left-Right Average

45. Left-Right Average

46. Swing Speed - Min - Left-Right Average

47. Swing Speed - Min - Left-Right Average

48. Left-Right Average

49. Step Frequency - Mean - Left-Right Average

50. Step Frequency - Mean - Left-Right Average

51. Left-Right Average

52. Step Frequency - Median - Left-Right Average

53. Step Frequency - Median - Left-Right Average

54. Left-Right Average

55. Step Frequency - Max - Left-Right Average

56. Step Frequency - Max - Left-Right Average

57. Left-Right Average

58. Step Frequency - Min - Left-Right Average

59. Step Frequency - Min - Left-Right Average

60. Left-Right Average

61. Stance Phase - Mean - Left-Right Average

62. Stance Phase - Mean - Left-Right Average

63. Left-Right Average

64. Stance Phase - Median - Left-Right Average

65. Stance Phase - Median - Left-Right Average

66. Left-Right Average

67. Stance Phase - Max - Left-Right Average

68. Stance Phase - Max - Left-Right Average

69. Left-Right Average

70. Stance Phase - Min - Left-Right Average

71. Stance Phase - Min - Left-Right Average

72. Left-Right Average

73. Swing Phase - Mean - Left-Right Average

74. Swing Phase - Mean - Left-Right Average

75. Left-Right Average

76. Swing Phase - Median - Left-Right Average

77. Swing Phase - Median - Left-Right Average

78. Left-Right Average

79. Swing Phase - Max - Left-Right Average

80. Swing Phase - Max - Left-Right Average

81. Left-Right Average

82. Swing Phase - Min - Left-Right Average

83. Swing Phase - Min - Left-Right Average

84. Left-Right Average

85. Step Height - Mean - Left-Right Average

86. Step Height - Mean - Left-Right Average

87. Left-Right Average

88. Step Height - Median - Left-Right Average

89. Step Height - Median - Left-Right Average

90. Left-Right Average

91. Step Height - Max - Left-Right Average

92. Step Height - Max - Left-Right Average

93. Left-Right Average

94. Step Height - Min - Left-Right Average

95. Step Height - Min - Left-Right Average

96. Left-Right Average

97. Step Width - Mean - Left-Right Average

98. Step Width - Median - Left-Right Average

99. Left-Right Average

100. Step Width - Max - Left-Right Average

101. Step Width - Min - Left-Right Average

102. Left-Right Average

103. Eyes-Closed Stance - Trunk Shake Degree

104. Eyes-Closed Stance - Trunk Forward Tilt Shake Degree

105. Eyes-Closed Stance - Trunk Left-Right Shake Degree

106. Eyes-Closed Stance - Total Time

107. Sit-to-Stand Test - Total Time

108. Sit-to-Stand Test - Actual Test Time

109. Sit-to-Stand Test - Turning Time

110. Sit-to-Stand Test - Stand Time

111. Sit-to-Stand Test - Sit Time

112. Sit-to-Stand Test - Step Speed

113. Repeated Sit-to-Stand - Total Time

114. Repeated Sit-to-Stand - Stand Speed

115. Repeated Sit-to-Stand - Stand Time

116. Repeated Sit-to-Stand - Sit Speed

117. Repeated Sit-to-Stand - Sit Time

118. Repeated Sit-to-Stand - Seat Stay Time

119. Repeated Sit-to-Stand - Trunk Shake Degree

120. Repeated Sit-to-Stand - Trunk Forward Tilt Shake Degree

121. Repeated Sit-to-Stand - Trunk Left-Right Shake Degree

122. Sit-to-Stand Test - Total Time

123. Sit-to-Stand Test - Actual Test Time

124. Sit-to-Stand Test - Turning Time

125. Sit-to-Stand Test - Stand Time

126. Sit-to-Stand Test - Sit Time

127. Sit-to-Stand Test - Step Speed

128. Tandem Stance - Total Time

129. Tandem Stance - Trunk Shake Degree

130. Tandem Stance - Trunk Forward Tilt Shake Degree

131. Tandem Stance - Trunk Left-Right Shake Degree

132. Half-Tandem Stance - Total Time

133. Half-Tandem Stance - Trunk Shake Degree

134. Half-Tandem Stance - Trunk Forward Tilt Shake Degree

135. Half-Tandem Stance - Trunk Left-Right Shake Degree
